# Stereotactic radiosurgery in the management of cluster headache: evidence from a systematic review and meta-analysis

**DOI:** 10.1007/s10143-025-04119-3

**Published:** 2026-01-31

**Authors:** Bardia Hajikarimloo, Salem M. Tos, Ibrahim Mohammadzadeh, Yuki Hannah Kim, Mohammad Amin Habibi, Yuki Shinya

**Affiliations:** 1 Department of Neurological Surgery, University of Virginia, Charlottesville, VA USA; 2 Skull Base Research Center, Loghman-Hakim Hospital, Shahid Beheshti University of Medical Sciences, Tehran, Iran; 3 School of Medicine, Tohoku University, Sendai, Japan; 4 Department of Neurosurgery, Shariati Hospital, Tehran University of Medical Sciences, Tehran, Iran

**Keywords:** Cluster headache, Radiosurgery, Stereotactic radiosurgery, Gamma knife, Meta-analysis

## Abstract

Cluster headache (CH) is a highly disabling primary headache disorder, and a subset of patients remain refractory to medical or neuromodulatory therapies. Stereotactic radiosurgery (SRS) has been explored as a minimally invasive alternative, but reported outcomes are inconsistent. A systematic review and meta-analysis was performed following PRISMA guidelines. A comprehensive search of PubMed, Embase, Scopus, and Web of Science from inception to September 15, 2025, identified eligible studies. Treatment techniques varied across studies, including radiosurgical targeting of the gasserian ganglion or the trigeminal nerve at different locations, with or without concomitant targeting of the sphenopalatine ganglion (SPG). Across five studies involving 51 patients, SRS showed a notable early benefit. The initial complete pain-free rate was 60.1% (95% CI: 24.4–91.3%), and the initial adequate relief rate was 80% (95% CI, 67.0–91.0%). At last follow-up before salvage, complete relief decreased to 28.8% (95% CI: 0–89.3%), and adequate relief to 41.7% (95% CI: 0.01–91.8%), demonstrating limited durability. The pain-recurrence rate was 59.8% (95% CI: 22.9–92.1%), and salvage therapy was required in 19% (95% CI: 7–34%). Adverse radiation effects (AREs) were common, and studies with longer follow-up reported high rates of permanent trigeminal sensory deficits, including anesthesia dolorosa, whereas studies with shorter follow-up likely underestimated late toxicity. SRS may provide early pain reduction in selected patients with medically refractory CH; however, the literature demonstrates a high rate of permanent trigeminal nerve injury, warranting cautious and highly selective use. SRS may serve as a selective or temporizing option when neuromodulatory therapies are not feasible. Limitations include small sample sizes, heterogeneous targets and dosimetry, and inconsistent definitions of outcomes. Future studies should employ prospective multicenter designs, standardized outcome metrics, optimized target planning, and extended follow-up to enhance patient selection.

**Supplementary information:**

The online version contains supplementary material available at 10.1007/s10143-025-04119-3.

## Introduction

Cluster headache (CH) is a rare but excruciating primary headache disorder belonging to the trigeminal autonomic cephalalgias (TACs) [1–3]. It is characterized by unilateral, severe, short-lasting attacks associated with ipsilateral autonomic features such as lacrimation, conjunctival injection, and rhinorrhea, often occurring with circadian and circannual periodicity [1–3]. The disease affects approximately 0.1% of the population, with chronic refractory forms causing profound disability, suicidality, and socioeconomic burden [1–3]. Pathophysiologically, CH involves dysfunction of the hypothalamus, activation of the trigeminovascular system, and overactivity of the parasympathetic outflow via the sphenopalatine ganglion (SPG), leading to the release of vasoactive neuropeptides such as calcitonin gene‑related peptide (CGRP) and pituitary adenylate cyclase-activating polypeptide 38 (PACAP-38) [1–3]. While acute treatments, such as oxygen and triptans, and preventive medications, including verapamil, lithium, and corticosteroids, remain first-line therapies, some patients remain refractory to pharmacologic management, necessitating the exploration of surgical and radiosurgical options [4].

Historically, ablative procedures involving the trigeminal nerve or SPG offered transient benefit but carried a high risk of facial numbness, dysesthesia, and deafferentation pain [5–9]. The introduction of stereotactic radiosurgery (SRS) provided a noninvasive alternative capable of lesioning deep neural structures with submillimetric precision [5–9]. Since Ford’s initial experience in 1998, subsequent studies have targeted the trigeminal nerve, SPG, or both, reporting heterogeneous results [5–9]. Early trials demonstrated limited long-term efficacy and high sensory morbidity. In contrast, more recent series have suggested improved safety and sustained pain relief with refined dose planning and dual-target strategies [5–9].

Despite these developments, the field remains constrained by small, non-comparative cohorts and variability in target selection, radiation dose, and outcome assessment. As a result, the overall efficacy, durability, and safety profile of this approach has not been clearly established. To address this gap, we performed a systematic review and meta-analysis of all published clinical studies of SRS for CH. Our objective was to summarize pooled rates of pain relief, recurrence, and adverse effects, and to explore factors that may influence treatment response.

## Materials and methods

### Objective

This study was conducted to evaluate the current role of SRS in the management of CH. The work followed the Preferred Reporting Items for Systematic Reviews and Meta-Analyses (PRISMA 2020) guidelines [10]. The study was not registered in any database or registry, given the exploratory nature and absence of prior meta-analysis.

### Search strategy

A comprehensive literature search was performed across PubMed, Embase, Scopus, and Web of Science, covering all available literature from database inception to September 15, 2025. The search strategy combined controlled vocabulary and free-text terms for “radiosurgery,” “stereotactic radiosurgery,” “Gamma Knife,” “CyberKnife,” and “cluster headache” or “trigeminal autonomic cephalalgia.” Detailed database-specific search strings are provided in Supplementary Table S1. No language or publication-type filters were applied.

### Eligibility criteria

The PICO framework used to define the scope of this systematic review is summarized in Supplementary Table S2. Studies were considered eligible if they met specific inclusion standards designed to capture high-quality evidence on SRS for CH. Eligible studies included clinical investigations reporting on patients with primary or secondary CH who were treated with SRS. Only cohorts with a minimum of five treated patients were included to ensure statistical reliability. Acceptable SRS modalities comprised Gamma Knife (GK) Radiosurgery, CyberKnife Radiosurgery, or Linear Accelerator platforms. Furthermore, studies were required to report at least one clinically relevant outcome, including pain relief, recurrence rates, or sensory adverse effects, and to be available in full-text English versions for detailed evaluation and data extraction. Studies were excluded if they did not meet these methodological or clinical thresholds. Specifically, case reports or series with fewer than five patients, non-SRS interventions, or studies that failed to distinguish CH outcomes from other headache disorders were omitted. Additionally, investigations lacking quantitative data that could be extracted, as well as animal studies, narrative reviews, editorials, and conference abstracts without full text, were excluded. Finally, duplicate or overlapping cohorts were identified and removed to prevent redundancy in pooled analyses.

### Study selection Process, data Extraction, and risk of bias assessment

All search results were imported into Covidence for systematic screening and removal of duplicates. Two reviewers independently conducted title and abstract screening, followed by a detailed evaluation of the full text of studies that met the preliminary eligibility criteria. Any discrepancies were resolved through consensus with a third senior reviewer to ensure methodological consistency. Data extraction was performed independently by two authors using a predefined, standardized template encompassing patient demographics, radiosurgical technique, and dosimetry (target site, maximum dose, and laterality), as well as pain outcomes, recurrence rates, and adverse events. The extracted variables and outcome definitions are provided in Supplementary Tables S3 and S4, respectively. The methodological quality and risk of bias of the included studies were evaluated using the Methodological Index for Non-Randomized Studies (MINORS) tool, which is specifically designed to assess both non-comparative and comparative clinical studies [11]. Each item was scored from 0 to 2, with higher totals reflecting superior methodological rigor. Two reviewers conducted Independent assessments, and any scoring inconsistencies were resolved by consensus with a third investigator.

### Statistical analysis

All analyses were performed in R using the meta and metafor packages. Proportions were pooled using the metaprop function with the Freeman–Tukey double arcsine transformation and inverse-variance weighting. Both fixed- and random-effects models were estimated, with between-study variance estimated by REML, and the Hartung–Knapp adjustment applied to random-effects confidence intervals. Heterogeneity was evaluated using Cochran’s Q and I²; values were considered notable when I² was greater than 40 or p was less than 0.10. Leave-one-out analyses were used to test model stability, and results were visualized in multi-panel forest plots. Primary outcomes were initial complete and adequate pain relief, and last follow-up complete and adequate pain relief before salvage. Secondary outcomes were pain recurrence and salvage intervention. Small-study effects were examined using Egger’s linear regression with a minimum of three studies and a trim-and-fill analysis. Meta-regression was done with logit-transformed proportions using REML estimation. Moderators included mean age, sex ratio, cluster subtype, symptom duration, target type, maximum dose, and follow-up duration. Statistical significance was set at *p* < 0.05.

## Results

### Study selection process

**Fig. 1:**
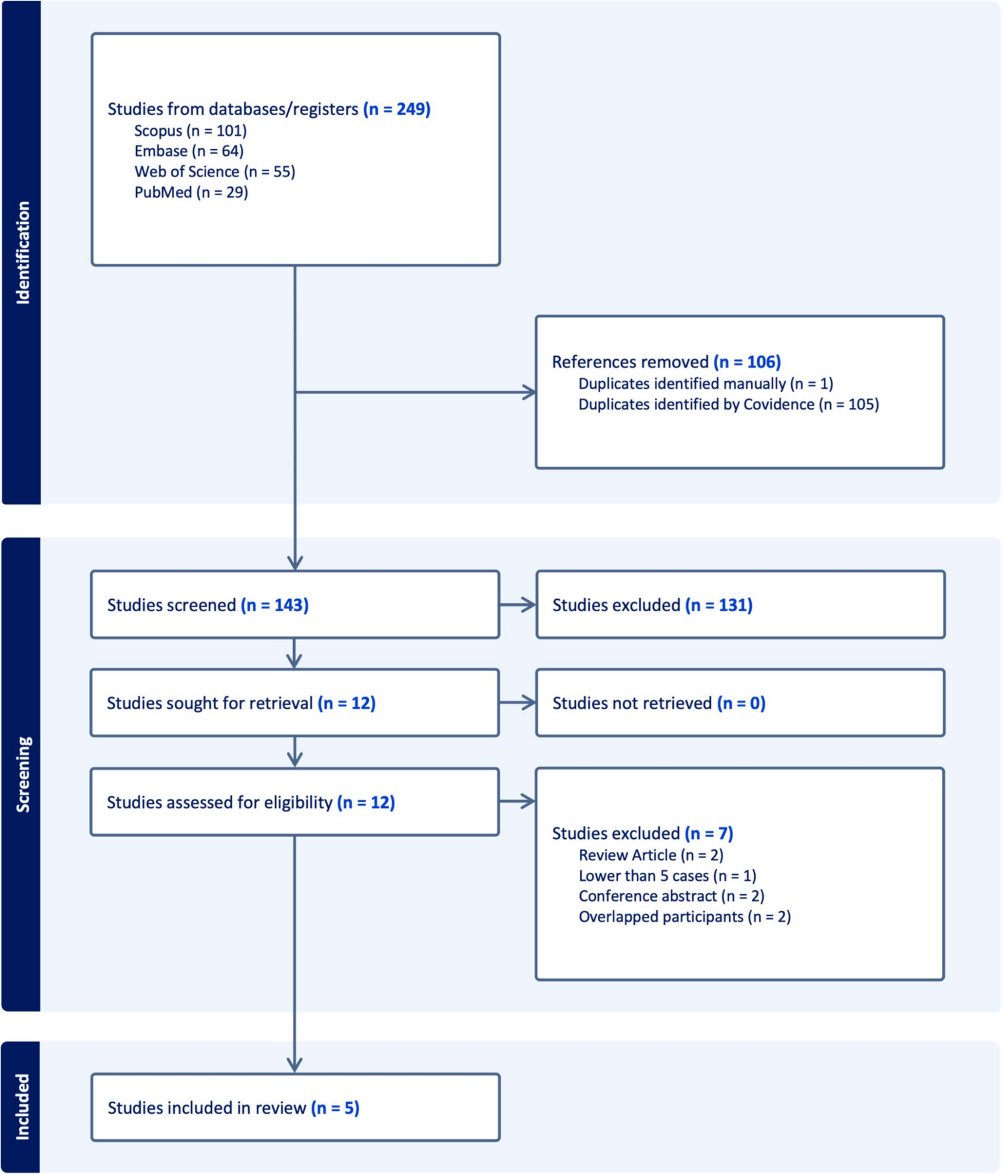
PRISMA flowchart of the included studies

A total of 249 records were identified through comprehensive database searches, including Scopus (*n* = 101), Embase (*n* = 64), Web of Science (*n* = 55), and PubMed (*n* = 29). Following removal of 106 duplicates (105 by Covidence and one by manual review), 143 studies remained for title and abstract screening. Of these, 131 were excluded as they did not meet the inclusion criteria, mainly due to irrelevant topic, non-SRS interventions, or lack of extractable clinical data. The remaining 12 full-text articles underwent detailed eligibility assessment. After this stage, seven studies were excluded: two were review articles, one involved fewer than five treated patients, two were conference abstracts without full text, and two had overlapping cohorts [12–17]. Ultimately, five studies fulfilled all predefined inclusion criteria and were incorporated into the quantitative synthesis [5–9]. The overall selection workflow is illustrated in Fig. 1.

### Risk of bias assessment

Across the included studies, methodological quality ranged from high to low, with total scores varying between 13 and 21 out of 21 (Supplementary Table S5). Overall, more recent investigations demonstrated stronger design rigor, with clearly defined inclusion criteria, standardized intervention protocols, comprehensive follow-up, and systematic outcome assessment, reflecting a low risk of bias. In contrast, earlier studies were limited by small sample sizes, lack of confounder control, and incomplete reporting, resulting in a moderate to high risk of bias. Collectively, the evidence suggests that while SRS for CH has been increasingly evaluated with improving methodological standards, early reports should be interpreted cautiously due to greater susceptibility to selection and reporting biases.

### Baseline characteristics

Five studies involving 51 CH patients treated with SRS were included in our study (Table 1). The included studies were published between 1998 and 2025, with 60% (3/5) being retrospective and 40% (2/5) being prospective. In the included studies, 60% (3/5) were conducted in the United States, 20% (1/5) were in Canada, and 20% (1/5) were in France. The mean age at SRS ranged from 40.3 to 52.6. The majority of the included cohort were males (92%, 46/51). The majority of the included patients had chronic cluster type (93.9%, 31/33). The mean duration of the symptoms ranged from 9 to 19 years. All patients were treated with SRS using GK. The primary SRS targets were the trigeminal nerve, SPG, or both. The maximum dose to the trigeminal nerve ranged from 70 to 88.8 Gy, and to the SPG from 80 to 90.3, depending on the treatment strategy.

**Table 1:** Baseline and SRS characteristics of patients with cluster headache treated with stereotactic radiosurgery

Study	Country	Patients	Mean Age (y)	Gender (M/F)	Cluster Type (Episodic/Chronic)	Symptom Duration (y, mean)	Prior Medical Tx (*n*)	Prior Surgical Tx (*n*)	SRS Use (Primary/Repeat)	SRS Platform	Head fixation	Target(s) (as reported)	Targets Treated (Trigeminal/SPG)	Max Dose to Trigeminal (Gy, mean)	Max Dose to SPG (Gy, mean)	Total SRS Procedures (*n*)
Mathieu et al., 2025	Canada	18	50.3	16/2	NA/NA	NA	18	NA	19/0	GKRS	Framed	Trigeminal Nerve, SPG	19/17	80.0	80	19
Ott et al., 2010	USA	7	52.6	7/0	1/6	19	7	NA	7/0	GKRS	Framed	Trigeminal Nerve, SPG	12/6	88.8	90.3	12
McClelland S 3rd, 2006	USA	10	40.3	10/0	0/10	11.3	10	NA	10/0	GKRS	Framed	Proximal cisternal trigeminal nerve ipsilateral to symptoms	10/0	75.0	NA	10
Donnet et al., 2006	France	10	49.8	9/1	0/10	9	10	0	10/0	GKRS	Framed	Trigeminal nerve (cisternal portion)	10/0	80.5	NA	10
Ford et al., 1998	USA	6	43.7	4/2	1/5	NA	6	0	6/0	GKRS	Framed	Trigeminal nerve (retrogasserian root entry zone)	6/0	70.0	NA	6

NA: Not available; M: Male; F: Female; Tx: Treatment; y: years; SRS: Stereotactic Radiosurgery; GKRS: Gamma Knife; SPG: Sphenopalatine ganglion; Gy: Gray

### Clinical and radiological outcomes

The mean follow-up in the included studies ranged from 10 to 48.1 months **(**Table 2**)**. Rates of initial complete pain relief varied widely, from 36.8% to 83.3%, while initial adequate pain relief ranged from 60% to 100%, indicating early efficacy in the majority of treated patients. At last follow-up before salvage, the rate of complete pain relief declined to 0–66.7%, and adequate pain relief persisted in 10–100%, reflecting partial loss of benefit over time, but with some patients maintaining long-term control. Pain recurrence occurred in 20–83.3% of patients across studies, whereas salvage interventions were required in 0–31.6%. Overall adverse radiation effects (AREs) were reported in 50–90%, although the majority were transient sensory disturbances (20–60%). Permanent AREs were infrequent (0–20%) and generally mild (paresthesia or hypoesthesia without anesthesia dolorosa).

**Table 2:** Outcome characteristics of patients with cluster headache treated with stereotactic radiosurgery

Study	Mean follow-up (months)	Initial complete pain relief	Initial adequate pain relief	Last FU complete pain relief before salvage	Last FU adequate pain relief before salvage	Pain recurrence before salvage	Salvage intervention	Overall ARE	Transient ARE	Permanent ARE	Initial complete facial numbness relief	Initial adequate facial numbness relief	Last FU complete facial numbness relief before salvage	Last FU adequate facial numbness relief before salvage
Mathieu et al., 2025	48.1	36.8%	78.9%	NA	15.8%	80%	31.6%	57.9%	10.5%	47.4%	94.4%	100%	52.6%	84.2%
Ott et al., 2010	10	80%	80%	60%	60%	25%	28.6%	80%	60%	20%	NA	NA	NA	NA
McClelland S 3rd, 2006	39.7	NA	60%	0%	10%	83.3%	NA	50%	0%	50%	NA	NA	50%	100%
Donnet et al., 2006	36.3	60%	80%	20%	30%	62.5%	10%	90%	20%	70%	0%	0%	10%	30%
Ford et al., 1998	11.7	83.3%	100%	66.7%	100%	20%	0%	50%	50%	0%	NA	NA	NA	NA

NA: Not available; ARE: Adverse Radiation Effect; FU: Follow-up; BNI: Barrow Neurological Institute; Percentages shown; counts omitted by design

### Meta-Analysis of outcomes

**Fig. 2:**
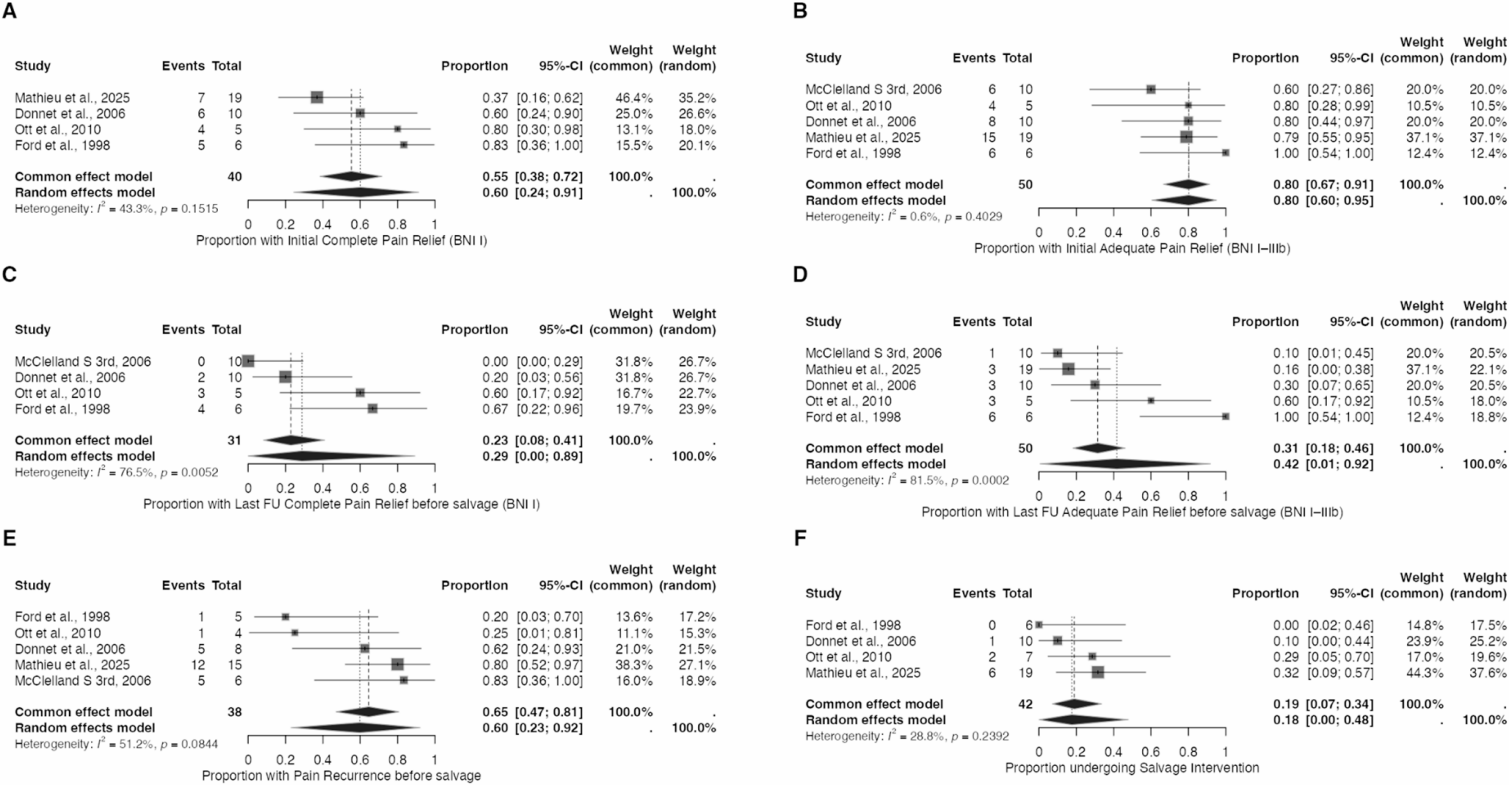
Leave-one-out meta-analyses of stereotactic radiosurgery outcomes. Each panel displays the pooled proportion and corresponding 95% confidence interval (CI) using random-effects and common-effect models for: **A** initial complete pain relief (BNI I); **B** initial adequate pain relief (BNI I–IIIb); **C** last follow-up complete pain relief before salvage (BNI I); **D** last follow-up adequate pain relief before salvage (BNI I–IIIb); **E** pain recurrence before salvage; and (**F**) salvage intervention rate. Diamonds represent pooled estimates, and the horizontal bars indicate 95% CIs. Heterogeneity statistics (I² and p values) are shown for each analysis

Pooled analysis across the included studies demonstrated meaningful rates of pain relief following SRS for CH **(**Fig. 2**)**. The initial complete pain-free rate was 60.1% (95% CI: 24.4–91.3%) under the random-effects model, with moderate heterogeneity (I² = 43%). The initial adequate pain-relief rate was 80% (95% CI: 67–91%), showing minimal heterogeneity (I² = 0.6%) and indicating consistent early benefit across studies. At last follow-up, before any salvage intervention, the pooled complete pain-free rate declined to 28.8% (95% CI: 0.0–89.3%), while the adequate pain-relief rate was 41.7% (95% CI: 0.01–91.8%). Both analyses demonstrated substantial heterogeneity (I² = 76.5% and 81.5%, respectively), reflecting variability in long-term durability among cohorts. The pain-recurrence rate before salvage was 59.8% (95% CI: 22.9–92.1%) (I² = 51.2%), indicating that recurrence after initial improvement remains a frequent occurrence. The salvage-intervention rate was 19% (95% CI: 0.07–34%), with low heterogeneity (I² = 28.8%).

Results of the meta-regression are summarized in Supplementary Table S6. Across outcomes, no consistent demographic or dosimetric predictor reached statistical significance for early response. However, longer follow-up duration was significantly associated with lower long-term pain-free rates (*p* = 0.011) and adequate pain-relief rates (*p* = 0.005), indicating that reported efficacy diminishes with time. The episodic subtype demonstrated greater sustained relief (*p* = 0.019) and a lower recurrence risk (*p* = 0.037) than the chronic subtype.

### Sensitivity analysis

Leave-one-out sensitivity analyses confirmed the stability and reliability of pooled estimates across all evaluated outcomes (Supplementary Figure S1). For initial complete and adequate pain relief, omitting any single study produced minimal change, supporting consistent early results. The last follow-up complete, and adequate pain-relief outcomes also remained directionally stable, with only minor fluctuations reflecting expected differences in follow-up duration. Likewise, the pain-recurrence and salvage-intervention analyses showed no meaningful deviation when studies were excluded. Collectively, these findings confirm that the meta-analytic results were robust and internally consistent across all efficacy and recurrence domains.

### Publication bias

Funnel plot inspection and Egger’s tests revealed no meaningful publication bias across outcomes (Supplementary Figure S3). A minor asymmetry was noted for initial complete pain relief (*p* = 0.019), but all other analyses showed non-significant results (*p* = 0.12–0.76). Trim-and-fill adjustments did not materially change the pooled estimates, confirming that the overall findings were not influenced by publication bias.

### GRADE assessment

The GRADE assessment showed overall moderate certainty for the primary outcome of initial adequate pain relief, supported by consistent findings and limited heterogeneity across studies. Certainty for initial complete pain-free response was low to moderate, downgraded due to imprecision and study limitations. For long-term outcomes at last follow-up, both complete and adequate pain relief demonstrated low certainty because of inconsistency and wide confidence intervals. Certainty for pain recurrence was low to moderate, and for salvage intervention was moderate, given the smaller heterogeneity and more precise estimates.

## Discussion

SRS has been explored for nearly three decades as a minimally invasive option for medically refractory CH, but its role remains controversial due to technical heterogeneity, limited durability, and sensory morbidity. This systematic review and meta-analysis synthesizes all available evidence across five cohorts, encompassing trigeminal nerve–only, dual-target trigeminal and SPG, and contemporary image-guided approaches, and provides pooled estimates of pain relief, durability, recurrence, and sensory complications. These findings clarify the position of SRS within the broader spectrum of neuromodulatory and lesioning therapies for refractory CH, including occipital nerve stimulation (ONS), SPG stimulation, and posterior hypothalamic deep brain stimulation (DBS).

Across 51 patients from five studies, SRS demonstrated strong early efficacy. The pooled initial complete pain-free rate was 60.1%, and the initial adequate pain-relief rate was 80%, indicating a reasonable early benefit. At last follow-up before salvage, complete pain relief declined to 28.8%, and adequate pain relief decreased to 41.7%, reflecting substantial variability and limited long-term durability. Pain recurrence occurred in 59.8%, and salvage therapy was required in 19%. Sensory AREs were documented in 50–90% of patients, with permanent symptoms in 0–20%, predominantly mild. Overall, SRS provides notable early improvement with acceptable toxicity, though long-term control remains inconsistent.

ONS has become one of the most established neuromodulatory treatments for medically refractory CH, supported by prospective and randomized studies demonstrating response rates of approximately 60–70% with sustained reductions in attack frequency and severity [18–22]. Meta-analytic data on occipital nerve–targeted interventions further support the role of the occipital pathway, with pooled short-term pain-free rates of 50% following occipital nerve blockade and generally mild, transient adverse effects [23, 24]. Compared with SRS, ONS offers greater long-term durability but requires implanted hardware and carries device-related risks; nonetheless, its reversible and non-destructive nature makes it an attractive option for patients seeking sustained benefit with lower risk of sensory or cranial nerve injury.

SPG stimulation is a targeted neuromodulatory therapy for medically refractory CH that exploits the trigeminal–autonomic reflex. In the pivotal randomized, double-blind trial, pain relief at 15 min was achieved in 62.5% of stimulation-treated attacks versus 38.9% with sham (OR 2.62, *p* = 0.008), confirming true physiological efficacy [25]. Long-term data show sustained benefit, with the 24-month CH-1 extension reporting 45% acute responders, 33% achieving ≥ 50% reduction in attack frequency, and 61% demonstrating either acute or preventive benefit [26]. Registry data from 97 patients showed similar outcomes, with 68% overall responders, 55% chronic-frequency responders, and 32% acute responders at 12 months, along with major reductions in medication use [27]. Prolonged remission has been reported in 30% of patients, with attack-free periods lasting a mean of 149 days beginning approximately 4–5 months after stimulation initiation [28]. Systematic reviews further support SPG stimulation, reporting abortive success rates of 67% and preventive responses in 35–50% of refractory patients, with adverse effects predominantly transient sensory disturbances and low infection risk [29, 30].

DBS of the posterior hypothalamic region is the most extensively studied invasive neuromodulatory therapy for refractory CH, supported by more than two decades of prospective, controlled, and meta-analytic data. An individual patient–data meta-analysis of 40 patients demonstrated a 77% (95% CI, 63–90%) reduction in headache frequency at a mean follow-up of 44 months, with a 75% responder rate, indicating durable long-term benefit [31]. A larger meta-analysis of 108 cases confirmed these findings, showing a 70.1% reduction in weekly attack frequency, a 50.6% decrease in intensity, and ≥ 50% improvement in frequency in 74.3% of patients [32]. Targeting has evolved from the posterior hypothalamus toward ventral and prerubral tegmental regions, where stimulation mapping has identified two midbrain hotspots associated with optimal analgesic response [31]. However, DBS carries substantial risk, including hardware complications, infection, diplopia, autonomic effects, and rare intracerebral hemorrhage, with a major complication rate of 16.7% [32]. Despite this, DBS remains the only intervention with strong evidence for long-term remission in the most treatment-resistant CH phenotypes, offering the highest durability among neuromodulatory options at the cost of greater procedural risk [33].

The pattern of early improvement followed by waning benefit likely reflects both biological and technical limitations of SRS in CH. Radiosurgical lesioning can transiently suppress trigeminal or SPG-mediated nociceptive transmission, producing early pain relief; however, long-term durability is poor, with recurrence commonly occurring within months to a few years. This pattern mirrors early trigeminal-only series, including the McClelland study, in which initial relief regressed in nearly all patients within two years [7], and the prospective Donnet trial, where short-term benefit was followed by relapse and high toxicity [8]. Given that CH is driven primarily by hypothalamic dysregulation rather than peripheral trigeminal pathology, SRS likely acts as a temporary modifier of pain pathways without altering the central generator of attacks, a conclusion further supported by the association between longer follow-up and lower pain-free rates in our meta-regression.

SRS offers a substantially safer profile than historical ablative procedures involving the trigeminal nerve or SPG. Surgical neuroablations, including rhizotomy, glycerol injection, and SPG sectioning, have historically produced high rates of anesthesia dolorosa, keratitis, facial weakness, and autonomic dysfunction. In contrast, across modern SRS series, adverse effects consist primarily of transient paresthesias, with permanent sensory deficits occurring at lower rates. Notably, two studies with longer follow-up reported high sensory toxicity rates (50–90%), substantially exceeding those typically reported after radiosurgery for trigeminal neuralgia, indicating that safety profiles are not directly comparable. Early series, such as those reported by Ford et al., even noted negligible short-term complications with no anesthesia dolorosa [9]. More contemporary dual-target studies, like the Ott et al. experience, similarly noted facial tingling without severe neuropathy or deafferentation pain [6]. Although medium- to long-term toxicity can be substantial when high trigeminal doses are used, as demonstrated by Donnet et al. (90% nerve disturbance, including deafferentation in some cases), these outcomes reflect older nerve-centered approaches rather than refined protocols with balanced brainstem dosing [8]. The more recent Mathieu et al. cohort reported bothersome numbness in only 16% after primary SRS, significantly lower than historical reports, underscoring technical progress in dose planning [5]. Overall, SRS remains far less morbid than open ablative surgery but requires meticulous dosimetry to avoid trigeminal over-irradiation.

These findings position SRS as a selective option for patients with refractory CH who decline invasive neuromodulation or are unsuitable for device implantation. SRS provides early pain reduction in most patients, with pooled initial improvement rates of 60–80%, but recurrence occurs in approximately 60–80% within 2–3 years, and durable complete pain freedom is uncommon. This distinguishes SRS from posterior hypothalamic DBS, which offers the highest long-term remission rates, and from SPG stimulation, which provides both acute and preventive effects. Accordingly, SRS may be best suited as a noninvasive, temporizing, or adjunctive therapy, particularly as a bridge to other neuromodulatory treatments, with modern dual-target approaches potentially offering a more favorable toxicity profile.

Lesioning strategies varied across studies, with some series targeting the trigeminal nerve alone at the cisternal or retrogasserian segment. In contrast, others employed dual targeting of the trigeminal nerve and the SPG, using different collimator sizes and dose prescriptions [5–9]. In long-term follow-up studies using trigeminal nerve–only targeting, durable pain relief was limited and high rates of trigeminal sensory morbidity, including deafferentation pain, were reported [7, 8]. Series incorporating dual targeting of the trigeminal nerve and SPG reported more consistent early pain relief, but recurrence remained common, and sensory adverse effects persisted, particularly after repeat SRS [5, 6]. Given the small cohorts and heterogeneity in targets, dose selection, and retreatment strategies, definitive conclusions regarding an optimal lesioning approach or dose cannot be drawn.

The evidence base for SRS in CH is limited by small sample sizes, heterogeneous study designs, and substantial variability in target selection, dose, and outcome reporting. Only five studies met the inclusion criteria, many of which used high trigeminal doses or nonstandard targets, complicating the interpretation of the pooled efficacy and toxicity. Most cohorts lacked standardized pain scales, consistent response definitions, or long-term follow-up, which contributed to wide confidence intervals and limited durability assessment. Although several series reported high toxicity and poor durability, these findings reflect heterogeneous, often outdated techniques that may not reflect contemporary practice. Additional confounders include potential publication bias, single-center designs, variation in cluster subtype and symptom duration, and differences in retreatment strategies, all of which likely influenced outcomes and toxicity.

Future work should prioritize prospective, multicenter studies with standardized outcomes to better define the role of SRS in CH. Larger cohorts are needed to determine whether dual-target strategies improve durability compared with trigeminal-only approaches and to optimize dose selection to reduce sensory morbidity. Advanced imaging and integration of SRS within multimodal treatment algorithms also warrant investigation to improve durability and patient selection.

## Conclusion

SRS provides meaningful early improvement in medically refractory CH, with initial complete and adequate pain relief rates of 60% and 80%, respectively. Although SRS can provide early pain reduction in selected patients, long-term durability is limited and sensory toxicity is more frequent than in trigeminal neuralgia; therefore, SRS should be considered a selective or temporizing option with careful patient counseling rather than a directly comparable alternative. Interpretation is constrained by the limited number of heterogeneous studies, variability in targets and doses, and inconsistent reporting of outcomes. Future multicenter prospective studies with standardized definitions, optimized dual-target planning, and longer follow-up are needed to clarify the true role of SRS in the contemporary treatment algorithm for refractory CH.

## Supplementary information

Below is the link to the electronic supplementary material.

Supplementary Material 1 (DOCX 22.9 KB)

Supplementary Material 2 (DOCX 341 KB)

## Data Availability

“The data supporting this study’s findings are available from the corresponding author upon reasonable request.”

## References

[1] Membrilla JA, Roa J, Díaz-de-Terán J (2023) Preventive treatment of refractory chronic cluster headache: systematic review and meta-analysis. J Neurol 270(2):689–710. 10.1007/s00415-022-11436-w 36310189 10.1007/s00415-022-11436-w

[2] Suri H, Ailani J (2021) Cluster headache: a review and update in treatment. Curr Neurol Neurosci Rep 21(7):31. 10.1007/s11910-021-01114-1 33948734 10.1007/s11910-021-01114-1

[3] Wei DY, Goadsby PJ (2021) Cluster headache pathophysiology - insights from current and emerging treatments. Nat Rev Neurol 17(5):308–24. 10.1038/s41582-021-00477-w 33782592 10.1038/s41582-021-00477-w

[4] Franzini A, Clerici E, Navarria P, Picozzi P (2022) Gamma knife radiosurgery for the treatment of cluster headache: a systematic review. Neurosurg Rev 45(3):1923–31. 10.1007/s10143-021-01725-9 35112222 10.1007/s10143-021-01725-9

[5] Mathieu D, Hamel A, Carrier L, Iorio-Morin C (2025) Stereotactic radiosurgery for cluster headache: A single center retrospective study. Neurosurgery [Internet]. ; Available from: 10.1227/neu.0000000000003725 10.1227/neu.0000000000003725 40932271

[6] Ott K, Hodgens DW, Goetsch S (2010) Gamma knife^®^radiosurgery of the trigeminal nerve and sphenopalatine ganglion for cluster headache. In: Radiosurgery [Internet]. Basel: KARGER; pp. 348–59. (Radiosurgery). Available from: 10.1159/000288745

[7] McClelland S, Tendulkar RD, Barnett GH, Neyman G, Suh JH (2006) Long-term results of radiosurgery for refractory cluster headache. Neurosurgery 59(6):1258–62. 10.1227/01.NEU.0000245614.94108.4B 17277688 10.1227/01.NEU.0000245614.94108.4B

[8] Donnet A, Tamura M, Valade D, Régis J (2006) Trigeminal nerve radiosurgical treatment in intractable chronic cluster headache: unexpected high toxicity. Neurosurgery 59(6):1252–7. 10.1227/01.NEU.0000245612.86484.7 17277687 10.1227/01.NEU.0000245612.86484.7

[9] Ford RG, Ford KT, Swaid S, Young P, Jennelle R (1998) Gamma knife treatment of refractory cluster headache. Headache 38(1):3–9. 10.1046/j.1526-4610.1998.3801003.x 9504996 10.1046/j.1526-4610.1998.3801003.x

[10] Page MJ, McKenzie JE, Bossuyt PM, Boutron I, Hoffmann TC, Mulrow CD et al (2021) The PRISMA 2020 statement: an updated guideline for reporting systematic reviews. PLoS Med 18(3):e1003583. 10.1371/journal.pmed.1003583 33780438 10.1371/journal.pmed.1003583 PMC8007028

[11] Slim K, Nini E, Forestier D, Kwiatkowski F, Panis Y, Chipponi J (2003) Methodological index for non-randomized studies (minors): development and validation of a new instrument. ANZ J Surg 73(9):712–6. 10.1046/j.1445-2197.2003.02748.x 12956787 10.1046/j.1445-2197.2003.02748.x

[12] Kano H, Kondziolka D, Niranjan A, Flickinger JC, Lunsford LD (2011) Γ knife stereotactic radiosurgery in the management of cluster headache. Curr Pain Headache Rep 15(2):118–23. 10.1007/s11916-010-0169-8 21181562 10.1007/s11916-010-0169-8

[13] Kano H, Kondziolka D, Mathieu D, Stafford SL, Flannery TJ, Niranjan A et al (2011) Stereotactic radiosurgery for intractable cluster headache: an initial report from the North American Gamma Knife Consortium. J Neurosurg 114(6):1736–43. 10.3171/2010.3.jns091843 20433278 10.3171/2010.3.JNS091843

[14] Kano H, Kondziolka D, Mathieu D, Stafford SL, Flannery TJ, Niranjan A et al (2009) Radiosurgery for cluster headache. Neurosurgery 65(2):417. 10.1227/01.neu.0000358721.62012.87

[15] McClelland S 3rd, Barnett GH, Neyman G, Suh JH (2007) Repeat trigeminal nerve radiosurgery for refractory cluster headache fails to provide long-term pain relief. Headache 47(2):298–300. 10.1111/j.1526-4610.2006.00701.x 17300376 10.1111/j.1526-4610.2006.00701.x

[16] Donnet A, Valade D, Régis J (2005) Gamma knife treatment for refractory cluster headache: prospective open trial. J Neurol Neurosurg Psychiatry 76(2):218–21. 10.1136/jnnp.2004.041202 15654036 10.1136/jnnp.2004.041202 PMC1739520

[17] Donnet A, Valade D, Régis J (2004) Trigeminal nerve radiosurgical treatment in intractable chronic cluster headache: Preliminary results. In: Radiosurgery [Internet]. Basel: KARGER; pp. 190–6. Available from: 10.1159/000078119

[18] Wilbrink LA, de Coo IF, Doesborg PGG, Mulleners WM, Teernstra OPM, Bartels EC et al (2021) Safety and efficacy of occipital nerve stimulation for attack prevention in medically intractable chronic cluster headache (ICON): a randomised, double-blind, multicentre, phase 3, electrical dose-controlled trial. Lancet Neurol 20(7):515–25. 10.1016/S1474-4422(21)00101-0 34146510 10.1016/S1474-4422(21)00101-0

[19] Fogh-Andersen IS, Sørensen JCH, Jensen RH, Knudsen AL, Meier K (2023) Treatment of chronic cluster headache with burst and tonic occipital nerve stimulation: a case series. Headache 63(8):1145–53. 10.1111/head.14617 37602914 10.1111/head.14617

[20] Burns B, Watkins L, Goadsby PJ (2007) Treatment of medically intractable cluster headache by occipital nerve stimulation: long-term follow-up of eight patients. Lancet 369(9567):1099–106. 10.1016/S0140-6736(07)60328-6 17398309 10.1016/S0140-6736(07)60328-6

[21] Magis D, Gerardy PY, Remacle JM, Schoenen J (2011) Sustained effectiveness of occipital nerve stimulation in drug-resistant chronic cluster headache. Headache 51(8):1191–201. 10.1111/j.1526-4610.2011.01973.x 21848953 10.1111/j.1526-4610.2011.01973.x

[22] Leone M, Proietti Cecchini A, Messina G, Franzini A (2017) Long-term occipital nerve stimulation for drug-resistant chronic cluster headache. Cephalalgia 37(8):756–63. 10.1177/0333102416652623 27250232 10.1177/0333102416652623

[23] Ornello R, Lambru G, Caponnetto V, Frattale I, Di Felice C, Pistoia F et al (2020) Efficacy and safety of greater occipital nerve block for the treatment of cluster headache: a systematic review and meta-analysis. Expert Rev Neurother 20(11):1157–67. 10.1080/14737175.2020.1809379 32781922 10.1080/14737175.2020.1809379

[24] Gordon A, Roe T, Villar-Martínez MD, Moreno-Ajona D, Goadsby PJ, Hoffmann J (2023) Effectiveness and safety profile of greater occipital nerve blockade in cluster headache: a systematic review. J Neurol Neurosurg Psychiatry 95(1):73–85. 10.1136/jnnp-2023-331066 36948579 10.1136/jnnp-2023-331066

[25] Goadsby PJ, Sahai-Srivastava S, Kezirian EJ, Calhoun AH, Matthews DC, McAllister PJ et al (2019) Safety and efficacy of sphenopalatine ganglion stimulation for chronic cluster headache: a double-blind, randomised controlled trial. Lancet Neurol 18(12):1081–90. 10.1016/S1474-4422(19)30322-9 31701891 10.1016/S1474-4422(19)30322-9

[26] Jürgens TP, Barloese M, May A, Láinez JM, Schoenen J, Gaul C et al (2017) Long-term effectiveness of sphenopalatine ganglion stimulation for cluster headache. Cephalalgia 37(5):423–34. 10.1177/0333102416649092 27165493 10.1177/0333102416649092 PMC5405839

[27] Barloese M, Petersen A, Stude P, Jürgens T, Jensen RH, May A (2018) Sphenopalatine ganglion stimulation for cluster headache, results from a large, open-label European registry. J Headache Pain [Internet]. ;19(1):6. Available from: 10.1186/s10194-017-0828-9 10.1186/s10194-017-0828-9 PMC5773459 29349561

[28] Barloese MCJ, Jürgens TP, May A, Lainez JM, Schoenen J, Gaul C et al (2016) Cluster headache attack remission with sphenopalatine ganglion stimulation: experiences in chronic cluster headache patients through 24 months. J Headache Pain. 10.1186/s10194-016-0658-1 27461394 10.1186/s10194-016-0658-1 PMC4961666

[29] Jürgens TP, May A (2014) Role of sphenopalatine ganglion stimulation in cluster headache. Curr Pain Headache Rep 18(7):433. 10.1007/s11916-014-0433-4 24880803 10.1007/s11916-014-0433-4

[30] Fontaine D, Santucci S, Lanteri-Minet M (2018) Managing cluster headache with sphenopalatine ganglion stimulation: a review. J Pain Res 11:375–81. 10.2147/JPR.S129641 29497328 10.2147/JPR.S129641 PMC5819579

[31] Nowacki A, Schober M, Nader L, Saryyeva A, Nguyen TAK, Green AL et al (2020) Deep brain stimulation for chronic cluster headache: Meta-analysis of individual patient data. Ann Neurol [Internet]. ;88(5):956–69. Available from: 10.1002/ana.25887 10.1002/ana.25887 32827225

[32] Murray M, Pahapill PA, Awad AJ (2023) Deep brain stimulation for chronic cluster headaches: a systematic review and meta-analysis. Stereotact Funct Neurosurg 101(4):232–43. 10.1159/000530508 37245509 10.1159/000530508

[33] Uwishema O, Shariff S, Chakik JAE, Bisetegn LD, Alomari O, Wojtara M (2025) Exploring the potential of deep brain stimulation in managing cluster headache: a systematic review. BMC Neurol 25(1):358. 10.1186/s12883-025-04373-4 40866814 10.1186/s12883-025-04373-4 PMC12382023

