# Supplementary material for: Stereotactic radiosurgery in the management of cluster headache: evidence from a systematic review and meta-analysis
Source: Neurosurg Rev. 2026 Jan 31;49(1):183. doi: 10.1007/s10143-025-04119-3 (PMC12860871; doi:10.1007/s10143-025-04119-3)
Supplement: Supplementary file 2 — Supplementary Material 2 (DOCX 341 KB) [file 10143_2025_4119_MOESM2_ESM.docx]

Supplementary File

Stereotactic Radiosurgery in the Management of Cluster Headache: Evidence from a Systematic Review and Meta-Analysis

| Supplementary Table S1: Search strategies for various databases. | |
| --- | --- |
| Database | Search strategy |
| PubMed | (“Radiosurgery"[MeSH Terms] OR radiosurg*[tiab] OR "stereotactic radiosurg*"[tiab] OR SRS[tiab]  OR "Gamma Knife"[tiab] OR GKRS[tiab] OR "CyberKnife"[tiab] OR CKRS[tiab]  OR LINAC[tiab] OR "linear accelerator"[tiab])  AND  (("Cluster Headache"[MeSH Terms] OR "cluster headache"[tiab] OR "trigeminal autonomic cephalalgia"[tiab])  OR (cluster[tiab] AND headache*[tiab])) |
| Embase | ('stereotactic radiosurgery'/exp OR radiosurg*:ti,ab OR 'stereotactic radiosurg*':ti,ab OR SRS:ti,ab  OR 'gamma knife':ti,ab OR GKRS:ti,ab OR 'cyberknife':ti,ab OR CKRS:ti,ab  OR LINAC:ti,ab OR 'linear accelerator':ti,ab)  AND  (('cluster headache'/exp OR 'cluster headache':ti,ab OR 'trigeminal autonomic cephalalgia':ti,ab)  OR (cluster:ti,ab AND headache*:ti,ab)) |
| Scopus | (TITLE-ABS-KEY(radiosurg* OR "stereotactic radiosurg*" OR SRS  OR "gamma knife" OR GKRS OR "cyberknife" OR CKRS OR LINAC OR "linear accelerator"))  AND  (TITLE-ABS-KEY("cluster headache" OR "trigeminal autonomic cephalalgia")  OR (TITLE-ABS-KEY(cluster) AND TITLE-ABS-KEY(headache*))) |
| WOS | TS=(radiosurg* OR "stereotactic radiosurg*" OR SRS  OR "gamma knife" OR GKRS OR "cyberknife" OR CKRS OR LINAC OR "linear accelerator")  AND  TS=("cluster headache" OR "trigeminal autonomic cephalalgia" OR (cluster AND headache*)) |
| September 15, 2025 | |

| Supplementary Table S2. PICO framework | |
| --- | --- |
| Population (P) | Patients with primary or secondary cluster headache (CH) who were refractory to conventional medical therapy and underwent stereotactic radiosurgery (SRS). |
| Intervention (I) | Stereotactic radiosurgery (SRS) performed using Gamma Knife Radiosurgery (GKRS), CyberKnife Radiosurgery (CKRS), or Linear Accelerator (LINAC) platforms, targeting the trigeminal nerve, sphenopalatine ganglion (SPG), or both. |
| Comparison (C) | No direct control group required |
| Outcome (O) | Primary outcomes: rates of initial and long-term pain relief, and pain recurrence. Secondary outcomes: rates of salvage interventions, sensory adverse effects, and treatment-related complications. |

| Supplementary Table S3. Extracted variables | |
| --- | --- |
| Baseline | Outcome |
| DOI, Study, Year, Country, Design, Full List of Participant Hospitals, Number of Patients, Number Treated with SRS, Mean Age, Number of Males, Number of Females, Number of Episodic Cluster Headache, Number of Chronic Cluster Headache, Mean Duration of Symptoms (years), Number with Prior Medical Treatment, Number with Prior Surgical Treatment, SRS Platform (GKRS, CKRS, LINAC), SRS Target (Trigeminal Nerve, Sphenopalatine Ganglion, Hypothalamus, Other), Number Targeted at Trigeminal Nerve, Number Targeted at Sphenopalatine Ganglion, Mean Maximum Dose to Trigeminal Nerve (Gy), Mean Maximum Dose to Sphenopalatine Ganglion (Gy), Immobilization Type (Frame, Frameless), Number of Primary SRS, Number of Repeat SRS, Mean Follow-up Duration (months). | Event Initial Complete Pain Relief (BNI I), Total Initial Complete Pain Relief (BNI I), Event Initial Adequate Pain Relief (BNI I–IIIb), Total Initial Adequate Pain Relief (BNI I–IIIb), Event Last Follow-up Complete Pain Relief (BNI I Before Salvage), Total Last Follow-up Complete Pain Relief (BNI I Before Salvage), Event Last Follow-up Adequate Pain Relief (BNI I–IIIb Before Salvage), Total Last Follow-up Adequate Pain Relief (BNI I–IIIb Before Salvage), Event Pain Recurrence Before Salvage, Total Pain Recurrence Before Salvage, Event Salvage Intervention, Total Salvage Intervention, Event Overall Adverse Radiation Effect, Total Overall Adverse Radiation Effect, Event Transient Adverse Radiation Effect, Total Transient Adverse Radiation Effect, Event Permanent Adverse Radiation Effect, Total Permanent Adverse Radiation Effect, Specification of ARE, Event Initial Complete Facial Numbness Relief (BNI I), Total Initial Complete Facial Numbness Relief (BNI I), Event Initial Adequate Facial Numbness Relief (BNI I–IIIb), Total Initial Adequate Facial Numbness Relief (BNI I–IIIb), Event Last Follow-up Complete Facial Numbness Relief (BNI I Before Salvage), Total Last Follow-up Complete Facial Numbness Relief (BNI I Before Salvage), Event Last Follow-up Adequate Facial Numbness Relief (BNI I–IIIb Before Salvage), Total Last Follow-up Adequate Facial Numbness Relief (BNI I–IIIb Before Salvage). |
|  |  |
|  |  |

| Supplementary Table S4. Definition of outcomes | |
| --- | --- |
| Initial Complete Pain Relief | Defined as complete resolution of cluster headache attacks (pain-free status) without the need for any acute or preventive medication following SRS. Corresponds to Barrow Neurological Institute (BNI) pain score I. |
| Initial Adequate Pain Relief | Defined as significant improvement or meaningful reduction in headache frequency or intensity, with or without limited use of medication (BNI I–IIIb), achieved during the initial post-treatment phase. |
| Last Follow-up Complete Pain Relief | Represents sustained complete pain relief (BNI I) at the final available follow-up before any salvage or repeat intervention was performed. |
| Last Follow-up Adequate Pain Relief | Defined as adequate or satisfactory pain control (BNI I–IIIb) maintained at last follow-up before salvage procedures or additional treatments. |
| Pain Recurrence | Defined as the reappearance or worsening of cluster headache attacks following an initial period of complete or adequate relief. |
| Salvage Intervention | Indicates the need for any secondary treatment (repeat SRS, surgical, or neuromodulatory procedure) due to recurrent or refractory pain after initial radiosurgery. |

| Supplementary Table S5. Risk of Bias Assessment | | | | | | | | |
| --- | --- | --- | --- | --- | --- | --- | --- | --- |
| Study | Confounding | Selection | Classification of interventions | Deviations from intended interventions | Missing data | Measurement of outcomes | Selection of reported result | Overall (Total /21) |
| Mathieu et al., 2025 | 3 | 3 | 3 | 3 | 3 | 3 | 3 | 21 / 21 (Low risk) |
| Ott et al., 2010 | 2 | 2 | 3 | 3 | 3 | 2 | 2 | 17 / 21 (Moderate risk) |
| McClelland S 3rd, 2006 | 2 | 3 | 3 | 3 | 3 | 2 | 3 | 19 / 21 (Moderate risk) |
| Donnet et al., 2006 | 2 | 3 | 3 | 3 | 3 | 2 | 2 | 18 / 21 (Moderate–high risk) |
| Ford et al., 1998 | 1 | 1 | 3 | 3 | 2 | 2 | 1 | 13 / 21 (High risk) |

| Supplementary Table S6. Meta-Regression Summary Table for SRS in Cluster Headache | | | | | | | |
| --- | --- | --- | --- | --- | --- | --- | --- |
| Outcome | Modifier | Coefficient | SE | Z | P_value | Tau2 | k |
| A_BNI Initial Complete | Mean age (y) | -0.129 | 0.196 | -0.659 | 0.510 | 0.519 | 4 |
| A_BNI Initial Complete | Male (%) | -0.026 | 0.055 | -0.478 | 0.632 | 0.639 | 4 |
| A_BNI Initial Complete | Female (%) | 0.026 | 0.055 | 0.478 | 0.632 | 0.639 | 4 |
| A_BNI Initial Complete | Episodic subtype (%) | 0.071 | 0.065 | 1.088 | 0.277 | 0.000 | 3 |
| A_BNI Initial Complete | Chronic subtype (%) | -0.071 | 0.065 | -1.088 | 0.277 | 0.000 | 3 |
| A_BNI Initial Complete | Targeted: Trigeminal nerve (%) | 0.014 | 0.021 | 0.678 | 0.498 | 0.541 | 4 |
| A_BNI Initial Complete | Targeted: SPG (%) | -0.010 | 0.011 | -0.852 | 0.394 | 0.476 | 4 |
| A_BNI Initial Complete | Max dose to Trigeminal (Gy) | -0.020 | 0.105 | -0.195 | 0.846 | 0.740 | 4 |
| A_BNI Initial Complete | Follow-up (months) | -0.055 | 0.025 | -2.248 | **0.025** | 0.000 | 4 |
| B_BNI Initial Adequate | Mean age (y) | 0.074 | 0.074 | 1.010 | 0.313 | 0.000 | 5 |
| B_BNI Initial Adequate | Male (%) | -0.059 | 0.043 | -1.372 | 0.170 | 0.000 | 5 |
| B_BNI Initial Adequate | Female (%) | 0.059 | 0.043 | 1.372 | 0.170 | 0.000 | 5 |
| B_BNI Initial Adequate | Episodic subtype (%) | 0.071 | 0.067 | 1.053 | 0.293 | 0.000 | 4 |
| B_BNI Initial Adequate | Chronic subtype (%) | -0.071 | 0.067 | -1.053 | 0.293 | 0.000 | 4 |
| B_BNI Initial Adequate | Symptom duration (y) | 0.023 | 0.140 | 0.167 | 0.867 | 0.066 | 3 |
| B_BNI Initial Adequate | Targeted: Trigeminal nerve (%) | 0.005 | 0.017 | 0.282 | 0.778 | 0.000 | 5 |
| B_BNI Initial Adequate | Targeted: SPG (%) | 0.004 | 0.007 | 0.508 | 0.611 | 0.000 | 5 |
| B_BNI Initial Adequate | Max dose to Trigeminal (Gy) | 0.026 | 0.079 | 0.332 | 0.740 | 0.000 | 5 |
| B_BNI Initial Adequate | Follow-up (months) | -0.016 | 0.028 | -0.580 | 0.562 | 0.000 | 5 |
| C_LastFU Complete (pre-salvage) | Mean age (y) | 0.127 | 0.193 | 0.659 | 0.510 | 2.250 | 4 |
| C_LastFU Complete (pre-salvage) | Male (%) | -0.053 | 0.060 | -0.879 | 0.379 | 1.761 | 4 |
| C_LastFU Complete (pre-salvage) | Female (%) | 0.053 | 0.060 | 0.879 | 0.379 | 1.761 | 4 |
| C_LastFU Complete (pre-salvage) | Episodic subtype (%) | 0.149 | 0.060 | 2.493 | **0.013** | 0.000 | 4 |
| C_LastFU Complete (pre-salvage) | Chronic subtype (%) | -0.149 | 0.060 | -2.493 | **0.013** | 0.000 | 4 |
| C_LastFU Complete (pre-salvage) | Symptom duration (y) | 0.221 | 0.185 | 1.193 | 0.233 | 1.079 | 3 |
| C_LastFU Complete (pre-salvage) | Targeted: Trigeminal nerve (%) | 0.020 | 0.028 | 0.730 | 0.465 | 2.027 | 4 |
| C_LastFU Complete (pre-salvage) | Targeted: SPG (%) | 0.017 | 0.023 | 0.730 | 0.465 | 2.027 | 4 |
| C_LastFU Complete (pre-salvage) | Max dose to Trigeminal (Gy) | 0.020 | 0.138 | 0.147 | 0.883 | 2.836 | 4 |
| C_LastFU Complete (pre-salvage) | Follow-up (months) | -0.090 | 0.036 | -2.536 | **0.011** | 0.000 | 4 |
| D_LastFU Adequate (pre-salvage) | Mean age (y) | 0.030 | 0.183 | 0.163 | 0.870 | 2.482 | 5 |
| D_LastFU Adequate (pre-salvage) | Male (%) | -0.092 | 0.064 | -1.440 | 0.150 | 1.291 | 5 |
| D_LastFU Adequate (pre-salvage) | Female (%) | 0.092 | 0.064 | 1.440 | 0.150 | 1.291 | 5 |
| D_LastFU Adequate (pre-salvage) | Episodic subtype (%) | 0.162 | 0.069 | 2.338 | **0.019** | 0.138 | 4 |
| D_LastFU Adequate (pre-salvage) | Chronic subtype (%) | -0.162 | 0.069 | -2.338 | **0.019** | 0.138 | 4 |
| D_LastFU Adequate (pre-salvage) | Symptom duration (y) | 0.161 | 0.164 | 0.980 | 0.327 | 0.757 | 3 |
| D_LastFU Adequate (pre-salvage) | Targeted: Trigeminal nerve (%) | 0.016 | 0.027 | 0.599 | 0.549 | 1.951 | 5 |
| D_LastFU Adequate (pre-salvage) | Targeted: SPG (%) | -0.004 | 0.019 | -0.228 | 0.820 | 2.662 | 5 |
| D_LastFU Adequate (pre-salvage) | Max dose to Trigeminal (Gy) | -0.038 | 0.140 | -0.271 | 0.787 | 2.532 | 5 |
| D_LastFU Adequate (pre-salvage) | Follow-up (months) | -0.074 | 0.026 | -2.817 | **0.005** | 0.000 | 5 |
| E_Pain Recurrence (pre-salvage) | Mean age (y) | -0.044 | 0.151 | -0.295 | 0.768 | 1.283 | 5 |
| E_Pain Recurrence (pre-salvage) | Male (%) | 0.051 | 0.052 | 0.987 | 0.324 | 0.724 | 5 |
| E_Pain Recurrence (pre-salvage) | Female (%) | -0.051 | 0.052 | -0.987 | 0.324 | 0.724 | 5 |
| E_Pain Recurrence (pre-salvage) | Episodic subtype (%) | -0.135 | 0.065 | -2.089 | **0.037** | 0.000 | 4 |
| E_Pain Recurrence (pre-salvage) | Chronic subtype (%) | 0.135 | 0.065 | 2.089 | **0.037** | 0.000 | 4 |
| E_Pain Recurrence (pre-salvage) | Symptom duration (y) | -0.181 | 0.159 | -1.139 | 0.255 | 0.347 | 3 |
| E_Pain Recurrence (pre-salvage) | Targeted: Trigeminal nerve (%) | -0.023 | 0.022 | -1.067 | 0.286 | 0.630 | 5 |
| E_Pain Recurrence (pre-salvage) | Targeted: SPG (%) | 0.002 | 0.015 | 0.159 | 0.874 | 1.351 | 5 |
| E_Pain Recurrence (pre-salvage) | Max dose to Trigeminal (Gy) | -0.007 | 0.113 | -0.061 | 0.951 | 1.236 | 5 |
| E_Pain Recurrence (pre-salvage) | Follow-up (months) | 0.073 | 0.027 | 2.663 | **0.008** | 0.000 | 5 |
| F_Salvage Intervention | Mean age (y) | 0.199 | 0.189 | 1.055 | 0.291 | 0.000 | 4 |
| F_Salvage Intervention | Male (%) | 0.043 | 0.051 | 0.851 | 0.395 | 0.051 | 4 |
| F_Salvage Intervention | Female (%) | -0.043 | 0.051 | -0.851 | 0.395 | 0.051 | 4 |
| F_Salvage Intervention | Episodic subtype (%) | 0.045 | 0.091 | 0.497 | 0.619 | 0.138 | 3 |
| F_Salvage Intervention | Chronic subtype (%) | -0.045 | 0.091 | -0.497 | 0.619 | 0.138 | 3 |
| F_Salvage Intervention | Targeted: Trigeminal nerve (%) | 0.009 | 0.017 | 0.492 | 0.623 | 0.356 | 4 |
| F_Salvage Intervention | Targeted: SPG (%) | 0.016 | 0.010 | 1.589 | 0.112 | 0.000 | 4 |
| F_Salvage Intervention | Max dose to Trigeminal (Gy) | 0.067 | 0.090 | 0.750 | 0.453 | 0.167 | 4 |
| F_Salvage Intervention | Follow-up (months) | 0.013 | 0.028 | 0.473 | 0.636 | 0.205 | 4 |


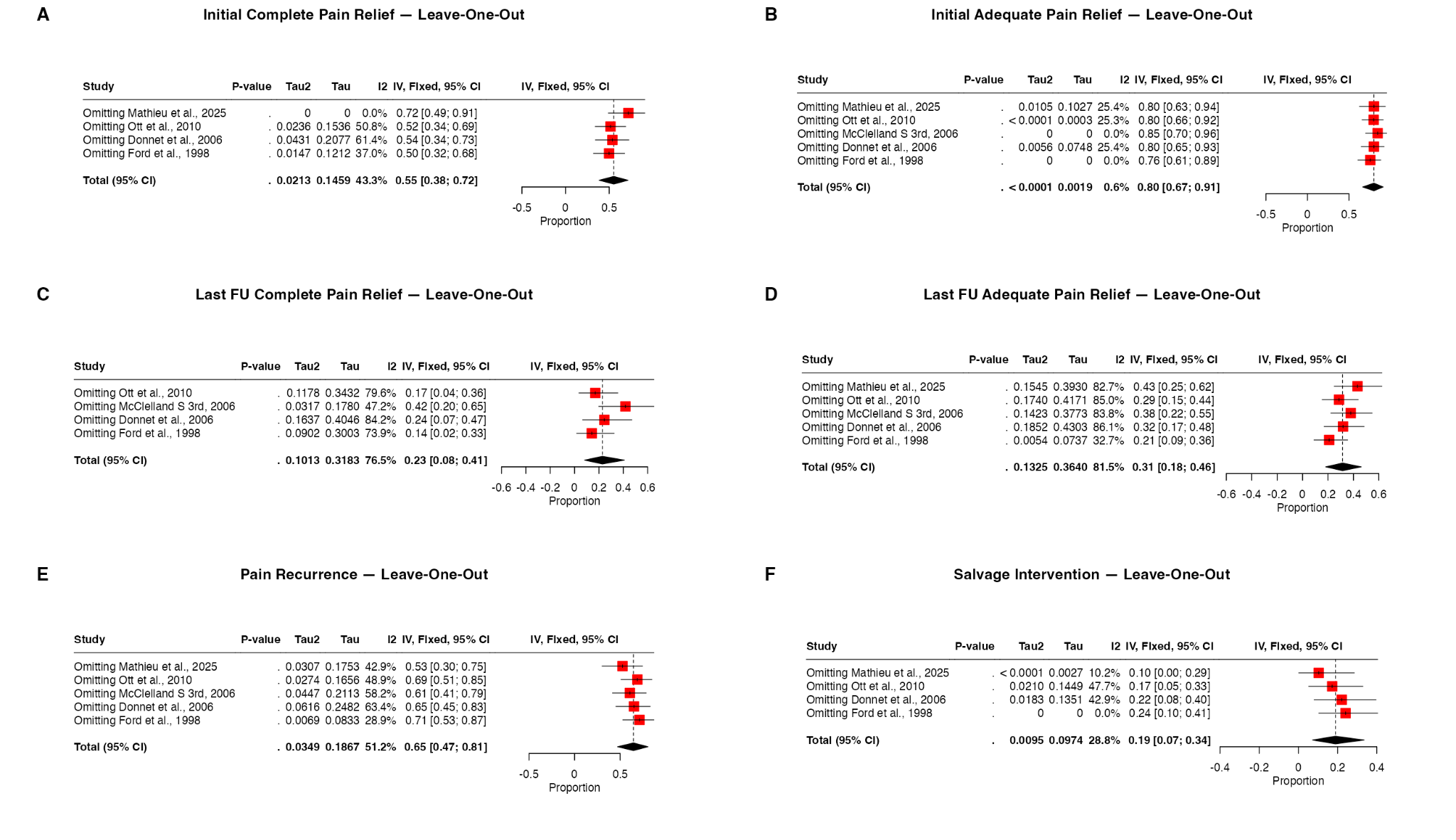


Supplementary Figure S1. Leave-one-out sensitivity analyses for all radiosurgical outcomes in cluster headache.
